# Development of Biodegradable Agar-Agar/Gelatin-Based Superabsorbent Hydrogel as an Efficient Moisture-Retaining Agent

**DOI:** 10.3390/biom10060939

**Published:** 2020-06-22

**Authors:** Jyoti Chaudhary, Sourbh Thakur, Minaxi Sharma, Vijai Kumar Gupta, Vijay Kumar Thakur

**Affiliations:** 1 School of Chemistry, Faculty of Sciences, Shoolini University, Solan, Himachal Pradesh 173229, India; jyotishuu26@gmail.com; 2 Center for Computational Materials Science, Institute of Physics, Slovak Academy of Sciences, 84511 Bratislava, Slovakia; 3 Department of Food Technology, Akal College of Agriculture, Eternal University, Baru Sahib, Himachal Pradesh 173101, India; minaxi86sharma@gmail.com; 4 AgroBioSciences (AgBS) and Chemical & Biochemical Sciences (CBS) Department, University Mohammed VI Polytechnic (UM6P), Lot 660, Hay Moulay Rachid, Benguerir 43150, Morocco; 5 Biorefining and Advanced Materials Research Center, Scotland’s Rural College (SRUC), Kings Buildings, West Mains Road, Edinburgh EH9 3JG, UK; 6 Department of Mechanical Engineering, School of Engineering, Shiv Nadar University, Uttar Pradesh 201314, India

**Keywords:** gelatin, agar-agar, green synthesis, hydrogel, biodegradable, swelling, soil, agriculture

## Abstract

Downgrading in the yield of crop is due to the inadequate availability of water. The way out for this trouble is to construct synthetic resources dependent on natural polymers with great water absorption and preservation limits. The present study investigated the design of agar-agar (Agr) and gelatin (GE) copolymerized methyl acrylate (MA) and acrylic acid (AA) hydrogel (Agr/GE-co-MA/AA) as a soil conditioner for moisture maintenance in agriculture. Agr/GE-co-MA/AA hydrogel was prepared by utilizing microwave-assisted green synthesis following the most suitable reaction conditions to obtain a remarkable water swelling percentage. The fabricated Agr/GE-co-MA/AA hydrogel was investigated through field emission scanning electron microscopy (FESEM), fourier transform infrared spectroscopy (FTIR), and X-ray diffraction (XRD). The water holding capacity of the soil and sand was examined by mixing Agr/GE-co-MA/AA hydrogel with soil and sand. The result demonstrates that the water holding time extended from 10 to 30 days for soil and 6 to 10 days for sand by using Agr/GE-co-MA/AA hydrogel. This synthesized biodegradable, low-cost and non-toxic Agr/GE-co-MA/AA hydrogel shows novelty as soil water maintaining material for irrigation in agriculture.

## 1. Introduction

**Scheme 1:**
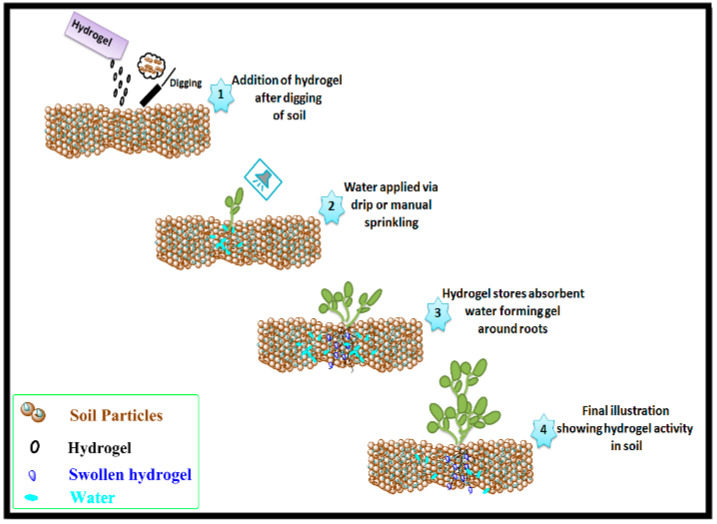
Demonstrates hydrogel used in the agriculture field for the function of plant water assimilation.

Sustainable development and growth in agriculture management of water is essential [1]. The inadequate supply of water is an exceedingly severe concern for agricultural activities [2]. Substitutes for the irrigation technique will become crucial in regions where the available water is decreasing with time [3]. Recently, absorbent material, like hydrophilic polymers, considers much concern for plant growth by maintaining the water and manure [4]. Hydrogels are characterized as cross-linked three-dimensional hydrophilic polymers, and have become one of the most recent interesting research topics due to their high swelling and hydrophilic nature [5]. Hydrogels are broadly recommended for agro purposes to remold water accessibility and swollen hydrogel stock water can be used for the function of plant water assimilation [6] as demonstrated in Scheme 1. 

Hydrogels can absorb and retain any biological fluid or aqueous media 100 times while correlating with the density [7]. The water absorption property depends upon the cross-linked arrangement and accessibility of hydrophilic units, like –OH, –SO_3_H, and –CONH_2_ [8]. The fascinating extremely large characteristic of water makes it a remarkable material that can be used in attractive goods, such as female hygiene sanitary, diapers, soil strengthener, drug delivery systems, and hydro blockade tapes [9,10,11,12]. Because of the superb water-absorbing tendency, absorbent materials are widely exposed to soil conditioning in the field. The utilization of water-sparing materials in farming for the recovery of infertile, barren, and aired fields requires splendid attempts and potential outcomes are initiated as they decrease the irrigation water utilization and the downfall of plants, increases the soil- grip for the fertilizer, and overhaul the plant upgrade standard [5]. 

The absorbent material probably affects the physical characteristics of the soil, i.e., upgrading the permeability rate of the soil, and diminishing soil corrosion and runoff. The integrated dry and wet techniques applied in soil decide the efficiency of hydrogel materials [13]. The absorbent materials are layered under the soil for reasonable execution. In cultivation, they are extensively used as moisture maintenance materials and bio-remediation purposes in soil. The manufacturing of advanced goods for spraying water strategies is significant for attaining lifelong beneficial evolution, generally in the regions where the accessibility of water is less [14].

Hydrogel materials from natural polymers build up an acceptable idea, being an abundant property, low-cost production, and biodegradable in nature [5]. Agar-agar (Agr) is a gelling agent, which acts as a remarkable soil water moisture-maintaining material and improves the harvest yield. Gelatin (GE) is acquired from animal bones and is soluble in lukewarm water, having low thickness properties in comparison with agar-agar [15]. Gelatin and agar-agar based materials have been exploited in various biomedical- and environmental-related applications; however, their utilization as a substitute for a water-retaining agent in the farming field still needs to be explored. 

Agricultural synthetic polymers are common but not biodegradable and their by-products are not eco-friendly [16]. Hydrogels made from Agr/GE can be utilized as an alternative material to synthetic polymers due to their biodegradability, non-toxicity, varying solubility, controlled release characteristics, and responsiveness to microbial degradation in the environmental atmosphere [17,18,19]. Table 1 provides a comparative chart of synthesized agar-agar (Agr) and gelatin (GE) copolymerized methyl acrylate (MA) and acrylic acid (AA) hydrogel (Agr/GE-co-MA/AA) absorbent material to that of other reported absorbents in the literature. The Agr/GE-co-MA/AA absorbent material is efficient compared to other reported materials from the water retention ability point of view. The easy fabrication, high absorption capacity, stability, and biodegradable nature make Agr/GE-co-MA/AA hydrogel an attractive alternative for irrigation treatment in the agriculture field. The present work concentrated on microwave-facilitated synthesis of Agr/GE-co-MA/AA hydrogel for the first time as a high moisture-retaining agent. 

The Agr/GE-co-MA/AA hydrogel swelling and water preservation properties were studied. The fabricated Agr/GE-co-MA/AA hydrogel was examined using fourier transform infrared spectroscopy (FTIR), X-ray diffraction (XRD), and field emission scanning electron microscopy (FESEM). The results of the water preservation studies demonstrate the excellent ability of Agr/GE-co-MA/AA hydrogel to maintain moisture in soil and sand for a long period. 

## 2. Materials and Methods 

### 2.1. Materials 

Gelatin (GE) (cattle bone), agar-agar (Agr), methyl acrylate (MA) (C_4_H_6_O_2_, 99%), acrylic acid (AA) (C_3_H_4_O_2_, 98%), *N*,*N*′-methylenebisacrylamide (NMBA) (C₇H₁₀N₂O₂, 99%), and ammonium persulfate (APS) ((NH₄)₂S₂O₈, 98%) were acquired from Loba Chemie, Central Drug House (Solan, India). Soil and sand were collected from the Himalayan region of North India (Shoolini University, Solan, Himachal Pradesh, India). Every single chemical was of analytical grade and utilized without future refinement. All reactions were completed utilizing deionized water.

### 2.2. Synthesis of Agar-Agar/Gelatin Copolymerized Methyl Acrylate/Acrylic Acid Hydrogel

The microwave-assisted method was used to synthesize Agr/GE-co-MA/AA. For a particular reaction, homogeneous solution of agar-agar/gelatin (1:1) was prepared in lukewarm water (temperature: 39 °C). In this solution, ammonium persulfate (0.24 mol L^−1^), methyl acrylate (1.10 × 10^−2^ mol L^−1^), acrylic acid (0.72 × 10^−2^ mol L^−1^), and *N*,*N*′-methylenebisacrylamide (0.16 mol L^−1^) were added. The mixture formed was stirred for 20 min at room temperature to ensure homogeneity of the reaction. After the formation of the homogeneous mixture, the mixture was placed in a microwave reactor (IFB Model-20PG3S microwave oven, 1000 W) operated at 20% microwave power for 90 s to complete the polymerization reaction. The successful polymerization reaction was confirmed by the appearance of gel-like material (Agr/GE-co-MA/AA). The formed Agr/GE-co-MA/AA hydrogel was washed with acetone to remove homopolymers and dried under a hot air oven at 50 °C for 24 h. Optimization of different reaction factors, like the initiator, monomer, solvent, cross-linker, time, and microwave power (Table 2) was carried out to obtain Agr/GE-co-MA/AA hydrogel with an excellent water absorption ability. 

### 2.3. Characterization

Fourier transform infrared spectroscopy (FTIR) interpretations were conducted using the KBr pellet method in the scale of 400–4000 cm^−1^ using a Nicolet 5700 FTIR spectrophotometer (Agilent Technologies, L1600312; CA, USA). The morphology of Agr/GE-co-MA/AA hydrogel was measured by a field emission scanning electron microscope (FESEM) (JSM-6100). The X-ray diffraction (XRD) was assessed by a SmartLab 9kW rotating anode x-ray (Rigaku Corporation, Tokyo, Japan). 

### 2.4. Swelling Studies of Agar-Agar/Gelatin Copolymerized Methyl Acrylate/Acrylic Acid Hydrogel

The pre-weighted Agr/GE-co-MA/AA hydrogel was taken in 40 mL of deionized water and kept for 18 h at room temperature. The swollen Agr/GE-co-MA/AA hydrogel was weighed on an analytical balance (accuracy: ±0.00001 g). The swelling percentage was evaluated through the following equation [22]:

(1) $$\%\;\mathrm{Swelling}=\frac{W_{s}-W_{d}}{W_{d}}\times 100,$$

where W_s_ is the weight of swollen Agr/GE-co-MA/AA hydrogel and W_d_ is the weight of dry Agr/GE-co-MA/AA hydrogel.

### 2.5. Water Retention Study

Two different soil and sand media were used to perform the water retention experiments by using Agr/GE-co-MA/AA hydrogel. In a particular experiment, 2 g of Agr/GE-co-MA/AA hydrogel was mixed with 20 g of soil taken in the plastic container. Then, 40 mL of tap water was slowly added into the plastic container and measured (W_1_) with an analytical balance (accuracy: ±0.00001 g). The control (soil without Agr/GE-co-MA/AA) experiment was also performed in a similar way (W_2_). The analysis containers were placed at room temperature and were weighed through an analytical balance daily until no clear decrease in weight was noted. The reduction in weight was measured daily with the help of an analytical balance (accuracy: ±0.00001 g). The water evaporation ratio (W %) of the soil was calculated through Equation (2) [15]:

(2) $$W\;\%\;=\frac{W_{1}-{\;W}_{2}}{40}\times 100$$

where W_1_ and W_2_ are the weights of the plastic container with Agr/GE-co-MA/AA hydrogel and without Agr/GE-co-MA/AA hydrogel, respectively; and 40 is the volume of water (mL). The same experiments for sand were repeated by using sand in place of soil. The FTIR was performed for Agr/GE-co-MA/AA hydrogel fragments after the final result of moisture retention to confirm their degradable nature.

## 3. Results and Discussions

**Scheme 2:**
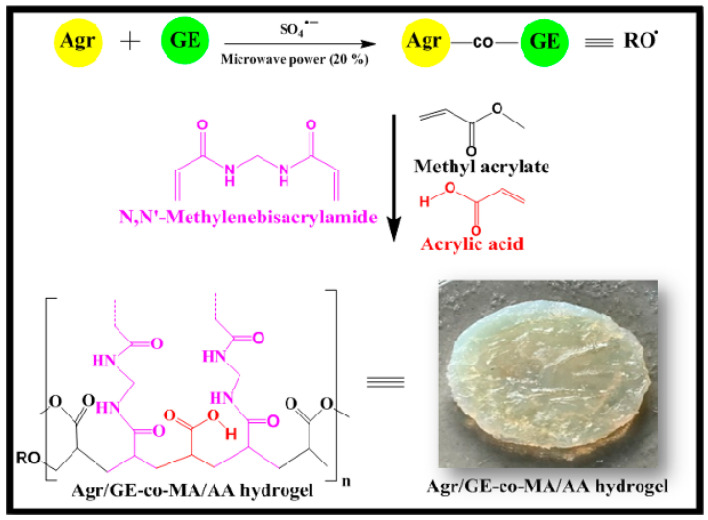
Representation of the synthesis of Agr/GE-co-MA/AA hydrogel.

The Agr/GE-co-MA/AA hydrogel was prepared by a microwave-assisted green method involving the polymerization reaction as presented in Scheme 2. 

In general, sulfate anion radicals are produced by disintegration of APS. Subsequently, the reaction of sulphate anion radicals with MA and AA initiates the reaction with the formation of monomer radicals. Meanwhile, the extraction of hydrogen from Agr/GE through SO_4_^●^ˉ (sulphate anion radicals) gives **^●^**O-R (alkoxy radicals). Thus, Agr/GE is converted to Agr/GE macro radicals followed by propagation steps. After the expansion of polymer chains, the monomer radicals that are closer to the reaction sites turn into acceptors of Agr/GE macroradicals. Accordingly, the cross-linked structure is formed during chain growth reactions by the concurrent reaction between polymer chains and vinyl groups of NMBA. Hydroxyl and other hydrophilic functional groups on Agr/GE contribute to the hydrogen bonding between Agr, GE, MA, AA, and NMBA, resulting in the formation of Agr/GE-co-MA/AA hydrogel. 

### 3.1. Optimization of Various Reaction Factors for the Swelling of Agar-Agar/Gelatin Copolymerized Methyl Acrylate/Acrylic Acid Hydrogel

**Figure 1:**
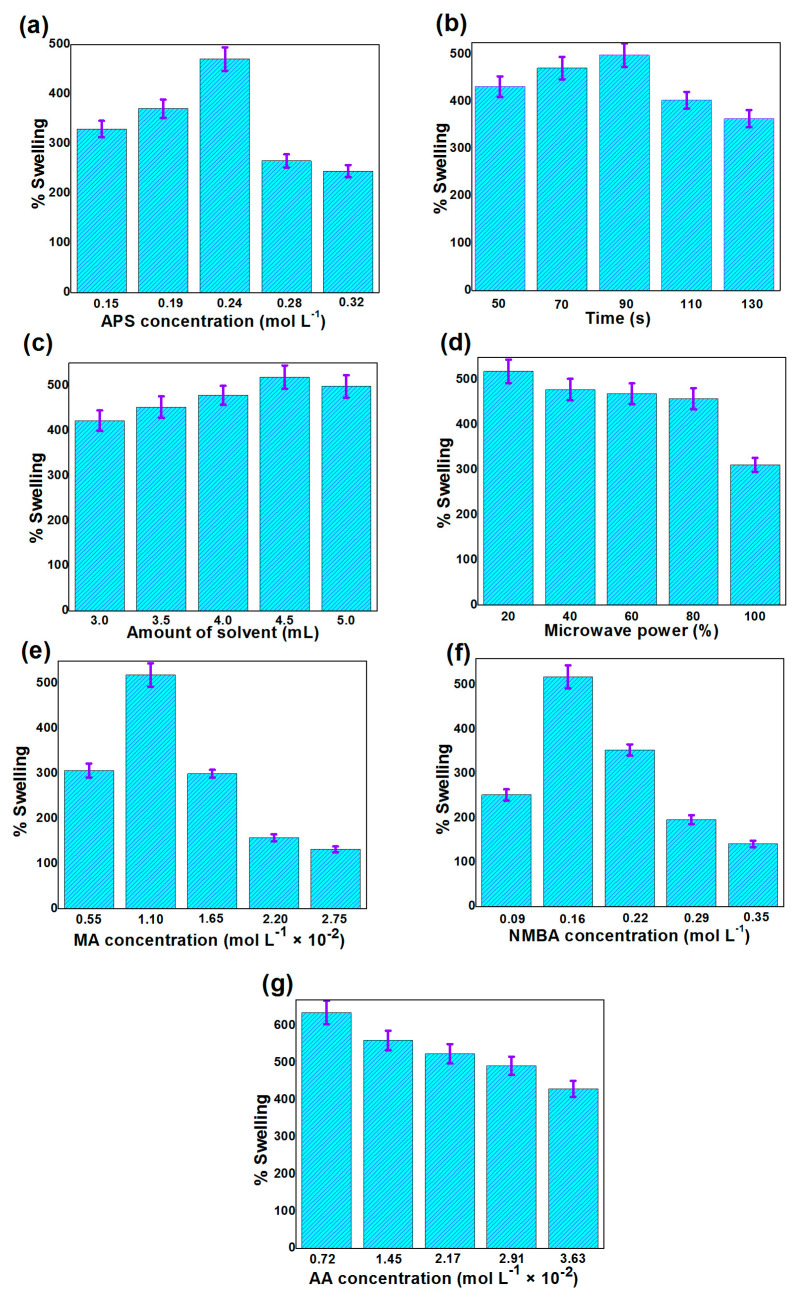
Swelling optimization of Agr/GE-co-MA/AA hydrogel as a function of (**a**) ammonium persulfate initiator, (**b**) reaction time, (**c**) solvent volume, (**d**) microwave power, (**e**) methyl acrylate concentration, (**f**) N, N′-methylenebisacrylamide concentration, and (**g**) acrylic acid concentration.

The outcome of the initiator concentration (APS) on the swelling percentage of Agr/GE-co-MA/AA hydrogel is shown in Figure 1a as the APS concentration varies in the range of 0.15–0.32 mol L^−1^. The % swelling rises with the increasing initiator concentration up to 0.24 mol L^−1^; subsequently, it begins to reduce. Initially, APS creates additional active sites on Agr/GE and MA/AA, resulting in the copolymerization process taking place rapidly, and leading to a rise in the percentage swelling. The higher initiator concentration support side chains and homo-polymerization processes, which are accountable for the reduction in the swelling percentage.

The effect of the reaction time on the swelling ability of Agr/GE-co-MA/AA hydrogel was analyzed in the range of 50–130 s and the outputs are presented in Figure 1b. The % swelling increases with an increase in the reaction time from 50 to 90 s, where it attains the maximum value. For the reaction time below 90 s, Agr/GE-co-MA/AA hydrogel shows lower % swelling, which can be ascribed to insufficient cross-linking, resulting in poor water uptake. However, when the reaction time is longer than 90 s, excessive cross-linking occurred, which results in a more rigid structure. This ultimately leads to a poor water uptake capability [23]. The solvent volumes utilized for the formation of Agr/GE-co-MA/AA hydrogel varied from 3 to 5 mL. The optimized value of the solvent is interpreted as 4.5 mL (Figure 1c). Approximately, the swelling percentage achieved at 4.5 mL of solvent was 518.7%. Further increments in the solvent volume led to a reduction in the swelling percentage because of a reasonable decrease in the concentration of the initiator (APS), monomer, and cross-linker (NMBA), which resulted in the poor polymerization rate [24]. The impact of the microwave power on the % swelling of Agr/GE-co-MA/AA hydrogel is presented in Figure 1d. The results demonstrated that the maximum % swelling was obtained at 20%, and thereafter, it decreased. With the increase in the microwave power, the dissociation rate of APS increased, which led to the formation of a large number of radicals, which further enhanced the homo-polymerization and termination reaction; hence, the swelling percentage decreased [25]. On increasing the monomer (MA) concentration from 0.55 × 10^−2^ mol L^−1^ to 1.10 × 10^−2^ mol L^−1^ (Figure 1e), the rate of copolymerization increased as more MA molecules were available for chain propagation sites and thus the swelling percent was enhanced. The decrease in the swelling percentage at a higher MA concentration (beyond 1.10 × 10^−2^ mol L^−1^) was due to the rise in the viscosity of the reaction, which prevented the movement of radicals and favored homo-polymerization over copolymerization [26]. The Agr/GE-co-MA/AA copolymer displayed the highest percentage swelling at a cross-linker (NMBA) concentration of 0.16 mol L^−1^ (Figure 1f). The decrease in percentage swelling with a further increase in the cross-linker concentration was due to the formation of a dense and inflexible Agr/GE-co-MA/AA hydrogel, which was not able to absorb a large amount of water [23]. The copolymer Agr/GE-co-MA/AA hydrogel shows a maximum swelling of 636.1% at the AA concentration of 0.72 × 10^−2^ mol L^−1^ (Figure 1g). 

### 3.2. Characterization 

#### 3.2.1. Fourier Transform Infrared Analysis 

**Figure 2:**
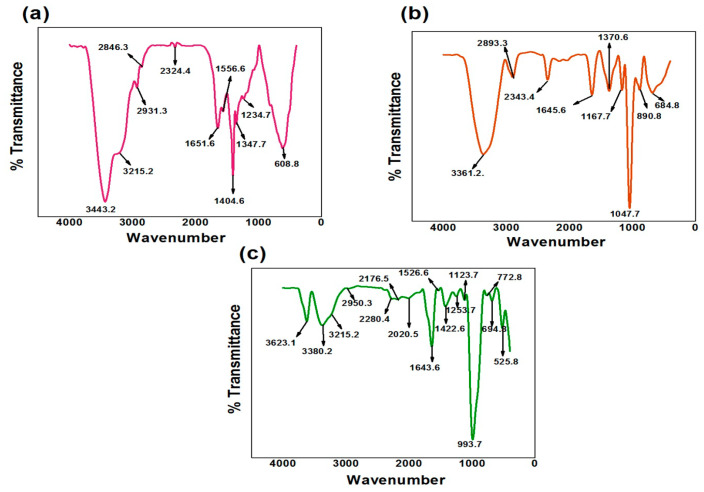
FTIR interpretations of (**a**) GE, (**b**) Agr, and (**c**) Agr/GE-co-MA/AA absorbent.

The FTIR interpretations of GE, Agr, and Agr/GE-co-MA/AA hydrogel were carried out to gain insight into the various interactions and functional groups (Figure 2). 

The FTIR interpretation of GE presented peaks around 3215.2, 3361.2, and 3443.2 cm^−1^ corresponding to N–H stretching of 2° amide, C=O stretching at 1651.6 cm^−1^, N–H bending between 1556.6 cm^−1^ and 1404.6 cm^−1^, N–H out of plane wagging at 608.8 cm^−1^, and C–H stretching at 928.2 and 2846.3 cm^−1^ [27] (Figure 2a). The Agr showed an intense and sharp peak at 1047.7 and 890.8 cm^−1^ related to 3,6-anhydro galactose networks and CH_3_ rocking, respectively [28] (Figure 2b). The characteristic adsorption band at 1643.6 cm^−1^ is ascribed to the >C=O stretching unit of AA [15] (Figure 2c). The peaks of MA at 2950.3 and 1526.6 cm^−1^ were due to C-H stretching vibration and C=O bonding correspondingly. Besides, the C-O-C stretching and deformation vibration were found at 1253.7 and 1123.7 cm^−1^, respectively, while peaks at 993.7, 772.8, 694.8, and 525.8 cm^−1^ were due to the C-H bending vibrations of polymer networks [29]. Some additional peaks (3380.2, 3215.2, 2950.3, 2280.4, 2176.5, and 2020.5 cm^−1^) and variation in intensity of peaks confirmed the copolymerization of MA, AA, Agr, and GE (Figure 2c).

#### 3.2.2. X-ray Diffraction 

**Figure 3:**
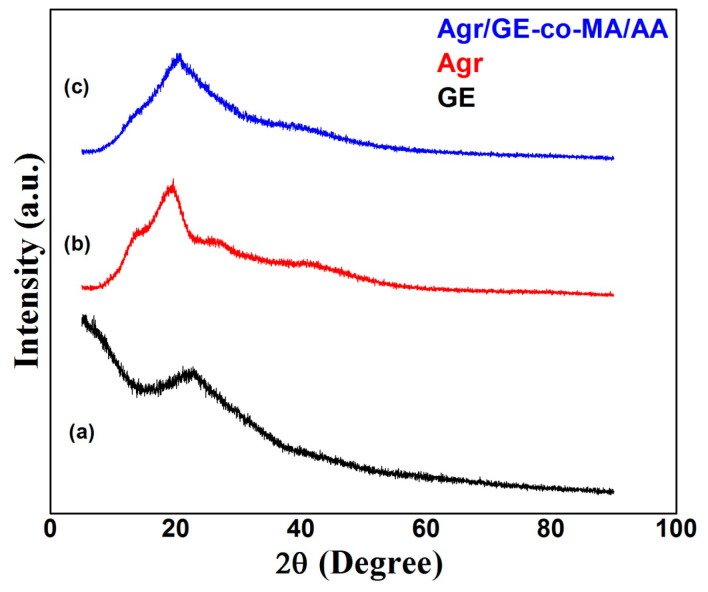
The XRD patterns of (**a**) GE, (**b**) Agr, and (**c**) Agr/GE-co-MA/AA hydrogel.

Gelatin shows a broad hump at 2θ ∼ 22.5 (Figure 3a). The XRD spectrum of Agr shows a peak at 2θ ∼ 19.1° with the intensity of 1624 (a.u), which demonstrates the semi-crystalline nature of Agr (Figure 3b). The intensity of the main diffraction peak decreased (relative intensity ∼1543) and shifted to the higher range (concerning Agr) (2θ ∼ 20.1°) in the case of Agr/GE-co-MA/AA hydrogel (Figure 3c). The broadening of the spectra proposed a change towards a disordered arrangement after copolymerization.

#### 3.2.3. Field Emission Scanning Electron Microscope 

**Figure 4:**
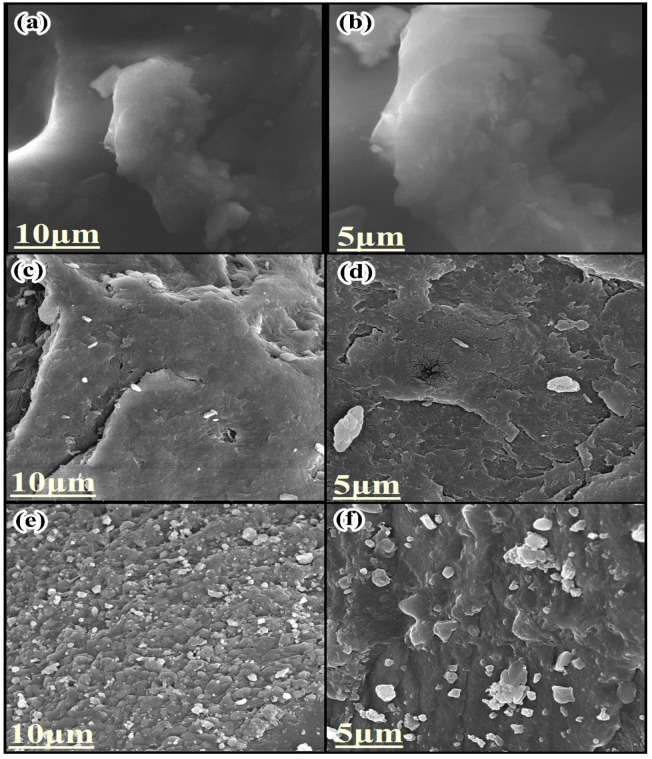
The FESEM morphology of (**a**,**b**) GE, (**c**,**d**) Agr, and (**e**,**f**) Agr/GE-co-MA/AA hydrogel at different magnifications.

The morphological characteristics of the Agr, GE, and Agr/GE-co-MA/AA hydrogel are displayed in Figure 4 through different magnification scales. The GE demonstrated a regular and uniform surface (Figure 4a,b). The Agr showed (Figure 4c,b) a smooth and compact surface morphology. The surface morphology of Agr/GE-co-MA/AA hydrogel changed into a distorted pattern with a rough and porous surface (Figure 4e,f). Hence, the Agr/GE-co-MA/AA hydrogel surface favored high water absorption. 

### 3.3. Water Retention Properties of Agar-Agar/Gelatin Copolymerized Methyl Acrylate/Acrylic Acid Hydrogel in Soil and Sand

**Figure 5:**
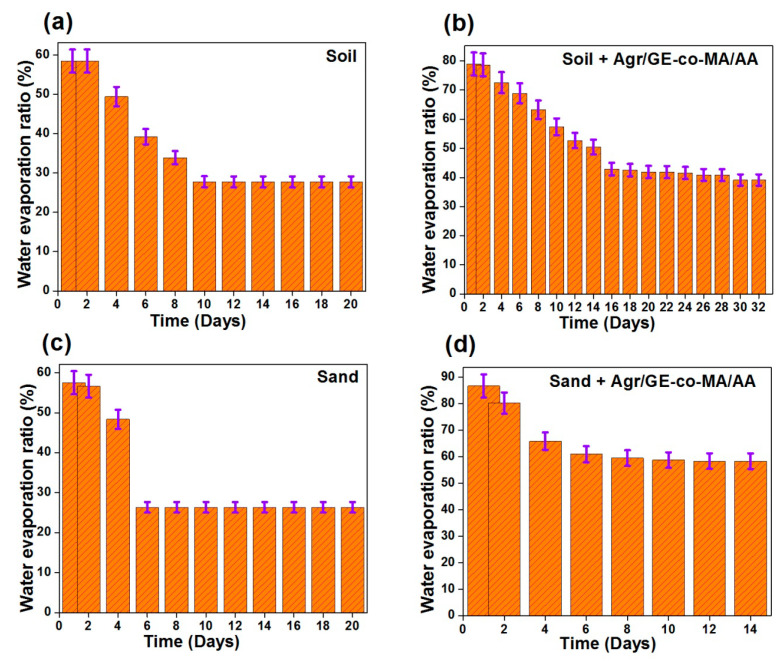
Water retention ratio of (**a**) soil, (**b**) Agr/GE-co-MA/AA hydrogel in soil, (**c**) sand, and (**d**) Agr/GE-co-MA/AA hydrogel in sand.

Nowadays, improvement in the water-holding ability of soil in cultivation is gaining extraordinary attention [16]. The pre-weighed Agr/GE-co-MA/AA hydrogel was used in soil to increase the water retention capacity. The water evaporation rate of soil and sand is shown in Figure 5. 

**Scheme 3:**
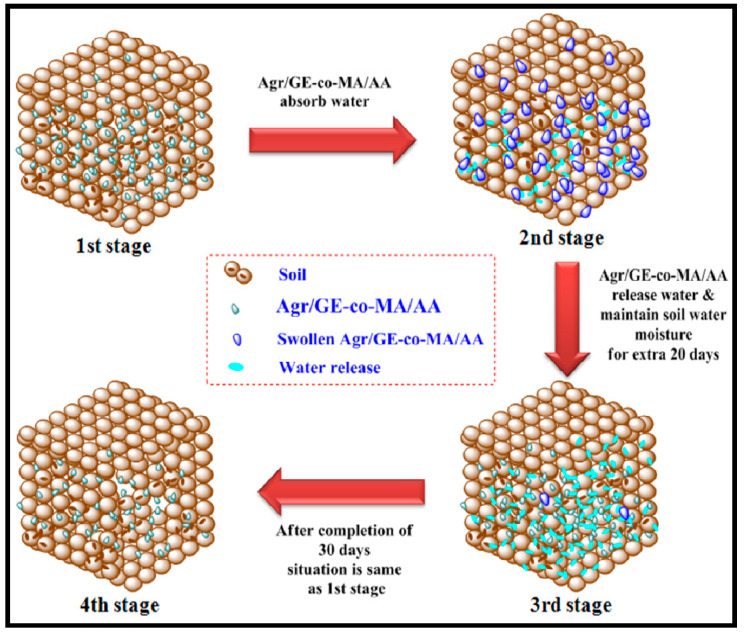
Schematic presentation of water retention in the soil through Agr/GE-co-MA/AA hydrogel.

The evaporation rate percentage in soil and sand containing Agr/GE-co-MA/AA hydrogel was less than that of the control (soil and sand without Agr/GE-co-MA/AA hydrogel). The rate of water loss was different for soil and sand, with variation in the number of days. The water evaporation levels in the soil and Agr/GE-co-MA/AA hydrogel in the soil are shown in Figure 5a,b. The water-retaining tendency for soil was increased three times when used with Agr/GE-co-MA/AA hydrogel. The sand with Agr/GE-co-MA/AA hydrogel was capable of holding the water as long as 10 days and water evaporated within 6 days in the case of sand only as presented in Figure 5c,d. The sandy loam soil can reduce moisture effortlessly in the atmosphere [30]. Thus, when Agr/GE-co-MA/AA hydrogel was sited with soil and sand, it upgraded the water-saving tendency (Scheme 3) up to 20 (soil) and 4 days (sand) extra because of the existence of a hydrophilic group, like –OH, –CONH, and –NH_2_, in the polymeric structure of Agr/GE-co-MA/AA hydrogel [15].

These outcomes show that the Agr/GE-co-MA/AA absorbent material can absorb and retain an immense amount of water [31]. The prepared Agr/GE-co-MA/AA hydrogel can be employed as a significant water-holding material in horticultural/agricultural applications.

### 3.4. Confirmation of Agar-Agar/Gelatin Copolymerized Methyl Acrylate/Acrylic Acid Hydrogel Biodegradability via Fourier Transform Infrared Study

**Figure 6:**
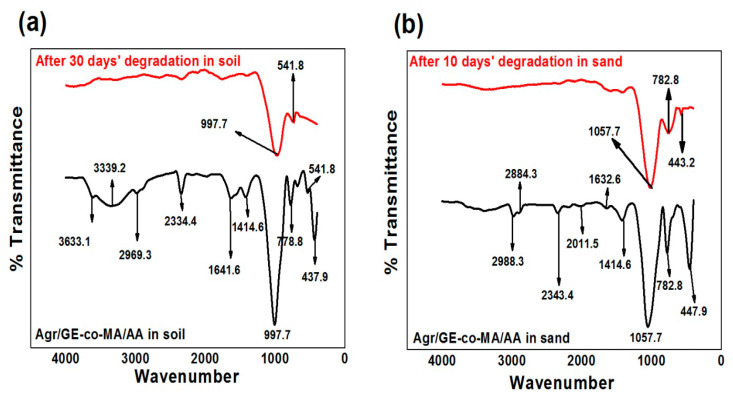
FTIR interpretation of Agr/GE-co-MA/AA hydrogel in (**a**) soil after 30 days and (**b**) sand after 10 days.

The FTIR results demonstrate that Agr/GE-co-MA/AA hydrogel begins degrading in the soil as well as in sand because of bacterial indigestion and bond breaking, mainly liable for H_2_O discharge (Figure 6).

The characteristic peaks at 3633.1, 3339.2, 2969.3, 2334.4, 1614.6, 1414.6, 2988.3, 2884.3, 2343.4, and 1414.6 cm^−1^ almost vanished and the transmittance values of the bands decreased. The cross-linked network of Agr/GE-co-MA/AA hydrogel with soil and sand led to the shifting of peaks and breaking of covalent bonds [32]. The degradation of Agr/GE-co-MA/AA hydrogel in soil and sand is a direct result of the activity of microbes and microorganisms. The degradation of fabricated Agr/GE-co-MA/AA hydrogel through bacterial indigestion demonstrates that it has no harmful impacts on sand and soil fertility and enhances the organic matter in the agricultural field.

## 4. Conclusions

A novel biodegradable Agr/GE-co-MA/AA superabsorbent was developed as an effective water-holding agent through microwave-supported synthesis. Agr/GE-co-MA/AA hydrogel exhibited a swelling capacity of 636.1% at optimized conditions (APS = 0.24 mol L^−1^, time = 90 s, solvent = 4.5 mL, microwave power = 20 %, MA = 1.10 × 10^−2^ mol L^−1^, NMBA = 0.16 mol L^−1^, AA = 0.72 × 10^−2^ mol L^−1^). The porous and rough surface of Agr/GE-co-MA/AA hydrogel favored the water retention ability of soil and sand. Agr/GE-co-MA/AA hydrogel was executed for water absorption in combination with sand and soil. Agr/GE-co-MA/AA was found to enhance the water absorption and retention ability of sand and soil effectively at a low dose of 2 g. The water-retaining capacity of soil and sand was improved up to three times and 1.6 times, respectively. Importantly, it is useful in an economic way, and is biodegradable and non-toxic. This biodegradable Agr/GE-co-MA/AA hydrogel can be applied as a promising absorbent as a water-holding agent in agricultural applications.

## Figures and Tables

**Table 1:** Comparison between water retention ratios of synthesized agar-agar/gelatin copolymerized methyl acrylate/acrylic acid (Agr/GE-co-MA/AA) hydrogel and reported absorbents.

S.N.*	Analysis of Media	Absorbent Material	Water Evaporation Ratio (Days)	Reference
1.	Soil	Alginate nanoparticles	11	[20]
2.	Sandy soil	Fenugreek galactomannan-borax hydrogel	11.5	[14]
3.	Alluvial soil	Nano clay polymer composite	13	[5]
4.	Soil	Double-coated, slow-release and water-retention urea fertilizer	17.3	[4]
5.	Clay soil	IPN hydrogel	27	[21]
6.	Soil	Agar/Ga-Cl-poly(AA)	28	[15]
7.	Sand	Agr/GE-co-MA/AA	10	Present work
8.	Soil	Agr/GE-co-MA/AA	30	Present work

* Serial number.

**Table 2:** Various reaction conditions for the synthesis of Agr/GE-co-MA/AA hydrogel.

S. N.	Initiator (APS) (mol L^−1^)	Reaction Time (s)	Solvent (mL)	Microwave power (%)	Monomer (MA) (mol L^−1^)	Crosslinker (NMBA) (mol L^−1^)	Monomer (AA) (mol L^−1^)	% Swelling
1.	0.15	70	5.0	20	1.10 × 10^−2^	0.16	--	329.6
2.	0.19	70	5.0	20	1.10 × 10^−2^	0.16	--	370.5
3.	0.24	70	5.0	20	1.10 × 10^−2^	0.16	--	470.7
4.	0.28	70	5.0	20	1.10 × 10^−2^	0.16	--	265.3
5.	0.32	70	5.0	20	1.10 × 10^−2^	0.16	--	244.9
6.	0.24	50	5.0	20	1.10 × 10^−2^	0.16	--	431.8
7.	0.24	70	5.0	20	1.10 × 10^−2^	0.16	--	470.7
8.	0.24	90	5.0	20	1.10 × 10^−2^	0.16	--	498.3
9.	0.24	110	5.0	20	1.10 × 10^−2^	0.16	--	402.9
10.	0.24	130	5.0	20	1.10 × 10^−2^	0.16	--	363.7
11.	0.24	90	3.0	20	1.10 × 10^−2^	0.16	--	422.2
12.	0.24	90	3.5	20	1.10 × 10^−2^	0.16	--	452.2
13.	0.24	90	4.0	20	1.10 × 10^−2^	0.16	--	478.5
14.	0.24	90	4.5	20	1.10 × 10^−2^	0.16	--	518.7
15.	0.24	90	5.0	20	1.10 × 10^−2^	0.16	--	498.3
16.	0.24	90	4.5	20	1.10 × 10^−2^	0.16	--	518.7
17.	0.24	90	4.5	40	1.10 × 10^−2^	0.16	--	478.1
18.	0.24	90	4.5	60	1.10 × 10^−2^	0.16	--	468.8
19.	0.24	90	4.5	80	1.10 × 10^−2^	0.16	--	458.1
20.	0.24	90	4.5	100	1.10 × 10^−2^	0.16	--	311.1
21.	0.24	90	4.5	20	0.55 × 10^−2^	0.16	--	306.8
22.	0.24	90	4.5	20	1.10 × 10^−2^	0.16	--	518.7
23.	0.24	90	4.5	20	1.65 × 10^−2^	0.16	--	299.6
24.	0.24	90	4.5	20	2.20 × 10^−2^	0.16	--	157.6
25.	0.24	90	4.5	20	2.75 × 10^−2^	0.16	--	132.2
26.	0.24	90	4.5	20	1.10 × 10^−2^	0.09	--	252.4
27.	0.24	90	4.5	20	1.10 × 10^−2^	0.16	--	518.7
28.	0.24	90	4.5	20	1.10 × 10^−2^	0.22	--	353.9
29.	0.24	90	4.5	20	1.10 × 10^−2^	0.29	--	196.4
30.	0.24	90	4.5	20	1.10 × 10^−2^	0.35	--	141.5
31.	0.24	90	4.5	20	1.10 × 10^−2^	0.16	0.72 × 10^−2^	636.1
32.	0.24	90	4.5	20	1.10 × 10^−2^	0.16	1.45 × 10^−2^	560.7
33.	0.24	90	4.5	20	1.10 × 10^−2^	0.16	2.17 × 10^−2^	525.0
34.	0.24	90	4.5	20	1.10 × 10^−2^	0.16	2.91 × 10^−2^	492.7
35.	0.24	90	4.5	20	1.10 × 10^−2^	0.16	3.63 × 10^−2^	430.2
